# Oxidative Stress in Aging

**DOI:** 10.1155/2014/876834

**Published:** 2014-04-06

**Authors:** Mohammad Abdollahi, Majid Y. Moridani, Okezie I. Aruoma, Sara Mostafalou

**Affiliations:** ^1^Faculty of Pharmacy and Pharmaceutical Sciences Research Center, Tehran University of Medical Sciences, Tehran 1417614411, Iran; ^2^Clinical Chemistry and Toxicology, Department of Pathology, Medical College of Wisconsin, 9200 W. Wisconsin Avenue, Milwaukee, WI 5322, USA; ^3^School of Pharmacy, American University of Health Sciences, Signal Hill, CA 90755, USA

Constant formation of free radicals mainly reactive oxygen species (ROS) is the main characteristic of all living systems which use oxygen for their basal metabolism. Primarily, ROS are considered an integral component of basic cell regulation and signaling pathways certainly, suppressing tumor progression which forms the basis of the most chemotherapeutic and radiotherapeutic agents. The most common ROS are superoxide radical (O_2_^·−^) and hydrogen peroxide (H_2_O_2_) which can stimulate consecutive reactions leading to further production of free radicals and related oxidative damage to cellular compartments. On the other side, there are several antioxidant complexes which defend cellular integrity against free radical-induced damages by neutralizing oxidative elements. Superoxide dismutase is the main antioxidant enzyme which converts O_2_^·−^ to H_2_O_2_ and then the other enzyme located in peroxisomes, catalase, disposes of H_2_O_2_ by converting it to O_2_ and H_2_O. Keeping the balance of such cell homeostasis in a level at which ROS are not able to damage cells is vital. Excessive ROS not only cause damage toward cellular lipids and DNA but also participate in posttranslational dysfunction of proteins or enzymes involved in keeping viability and function of the cells [1, 2]. Accordingly, oxidative stress and the accompanying pathway, inflammation, are known to be involved in the pathology of numerous human diseases including cancer, neurodegenerative diseases, rheumatoid arthritis, and diabetes. As an example, oxidative and inflammatory mechanisms were considered for neurodegenerative diseases primarily when it was observed that using high amounts of anti-inflammatory drugs in rheumatoid arthritis patients for long times was associated with less incidence of Alzheimer's disease (AD). However, there are many factors affecting the rate of ROS formation in the body among which lifestyle related habits like diet, cigarette smoking, alcohol consumption, environmental exposures, and stress are the most important. Apart from all these factors, it is evident that ROS and the other free radicals are more produced and accumulated in the body over time. This has led to a hypothesis on the implication of oxidative stress in aging and inspired researchers to find new therapies for battling age-related diseases through exploiting agents which modulate oxidant/antioxidant homeostasis [1, 2]. This special issue is composed of articles focused on describing the seminal research and viewpoints pertinent to current understanding of the role of oxidative stress in aging and perspectives on treatment of age-related diseases.

In this special issue, the comparative effects of biodynes, tocotrienol-rich fraction, and tocopherol in enhancing collagen synthesis and inhibiting collagen degradation in stress-induced premature senescence model of human diploid fibroblasts have been described. Skin aging can be intrinsic or extrinsic, mediated genetically or due to environmental exposures. Biodynes, tocotrienol-rich fraction, and tocopherol upregulate collagen genes and increase the synthesis of procollagen proteins.

The AD is the outcome of a complex interaction among several factors which are not fully understood yet; nevertheless, it is clear that oxidative stress and inflammatory pathways are among these factors. In this special issue, lower plasmatic levels of *α*-tocopherol and mild systemic oxidative stress in the subclinical stage of Alzheimer's disease have been described.

Also it has been described that sublethal oxidative stress could induce premature senescence of human mesenchymal stem cells derived from endometrium. Thus induction of premature senescence might be a common physiological response to sublethal oxidative stress in human mesenchymal stem cells of any origin.

Carbon monoxide (CO), an endogenous small gaseous mediator, may exert important roles in physiological and pathophysiological states through regulation of cellular signaling pathways. It has been noted that CO when applied at low concentration can confer anti-inflammatory effects in macrophages and protect endothelial cells and hepatocytes against cytotoxic agents.

The relationship between oxidative stress and aging by addressing cellular expression profile analysis through proteomics studies using two-dimensional electrophoresis and mass spectrometry has been used as an integral approach to study the aging process. Emphasis is placed on postmitotic tissues, such as neuronal, muscular, and red blood cells, which appear to be those most frequently studied with respect to aging.

Ovarian aging-like phenotype in the hyperandrogenism-induced murine model of polycystic ovary (PCO) is a new topic. Exposure to some environmental factors and chemicals during life may accelerate progression towards the end of functional reproductive period. Hyperandrogenism-induced PCO in rats is associated with ovarian aging-like phenotypes. Meanwhile, hyperandrogenism triggers ovarian senescence in rats with PCO.

Also in this special issue it was noted that increased oxidative stress response in granulocytes from older patients with a hip fracture may account for slow regeneration. Aged granulocytes seem more sensitive towards damage induced by oxidative stress rather than young granulocytes, declaring why aged granulocytes cannot cope with the additional oxidative stress stimuli of the fracture.

The production of ROS is not limited to a specific tissue or organ and oxidative based damages to the cells can be detected from the most superficial layer, skin, to different internal compartments like nervous system, bone marrows and hematopoietic system, liver, and the other parts of endocrine system. Protective effects of antioxidative agents like *α*-tocopherol and ascorbic acid in decreasing the rate of senescence have been well studied, of course, mostly in experimental models. However, effectiveness of modulators of oxidative homeostasis in treatment of age-related diseases still needs more studies along with opening more windows from the interactions of oxidative elements in aging [3]. The collection of data brought in this special issue is anticipated to help researchers to resolve key questions about the role of oxidative stress in aging and find promising perspectives on the efficacy of oxidative modulating agents in treatment or controlling age-related diseases.

*Mohammad Abdollahi* *Mohammad Abdollahi* *Majid Y. Moridani* *Majid Y. Moridani* *Okezie I. Aruoma* *Okezie I. Aruoma* *Sara Mostafalou* *Sara Mostafalou*
